# Lipase and biosurfactant from *Ochrobactrum intermedium* strain MZV101 isolated by washing powder for detergent application

**DOI:** 10.1186/s12944-017-0565-8

**Published:** 2017-09-18

**Authors:** Mina Zarinviarsagh, Gholamhossein Ebrahimipour, Hossein Sadeghi

**Affiliations:** Department of Microbiology and Microbial Biotechnology, Faculty of Biological Sciences and Technology, University of Shahid–Beheshty, Daneshjou Blvd. Evin St.1983969411, Tehran, Iran

**Keywords:** Lipase, Biosurfactant, *Ochrobactrum intermedium*, Washing powder

## Abstract

**Background:**

Alkaline thermostable lipase and biosurfactant producing bacteria are very interested at detergent applications, not only because of their eco-friendly characterize, but alsoproduction lipase and biosurfactant by using cheap materials. *Ochrobactrum intermedium* strain MZV101 was isolated as washing powder resistant, alkaline thermostable lipase and biosurfactant producing bacterium in order to use at detergent applications.

**Methods:**

*O. intermedium* strain MZV101 produces was lipase and biosurfactant in the same media with pH 10 and temperature of 60 °C. Washing test and some detergent compatibility character of lipase enzyme and biosurfactant were assayed. The antimicrobial activity evaluated against various bacteria and fungi.

**Results:**

Lipase and biosurfactant produced by *O. intermedium* strain MZV101 exhibited high stability at pH 10–13 and temperature of 70–90 °C, biosurfactant exhibits good stability at pH 9–13 and thermostability in all range. Both lipase and biosurfactant were found to be stable in the presence of different metal ions, detergents and organic solvents. The lipase enzyme extracted using isopropanol with yield of 69.2% and biosurfactant with ethanol emulsification index value of 70.99% and yield of 9.32 (g/l). The single band protein after through from G-50 Sephadex column on SDS-PAGE was calculated to be 99.42 kDa. Biosurfactant *O. intermedium* strain MZV101 exhibited good antimicrobial activity against Gram-negative bacteria and against various bacterial pathogens. Based upon washing test biosurfactant and lipase *O. intermedium* strain MZV101considered being strong oil removal.

**Conclusion:**

The results of this study indicate that isolated lipase and biosurfactant with strong oil removal, antimicrobial activity and good stability could be useful for detergent applications.

**Graphical abstract:**

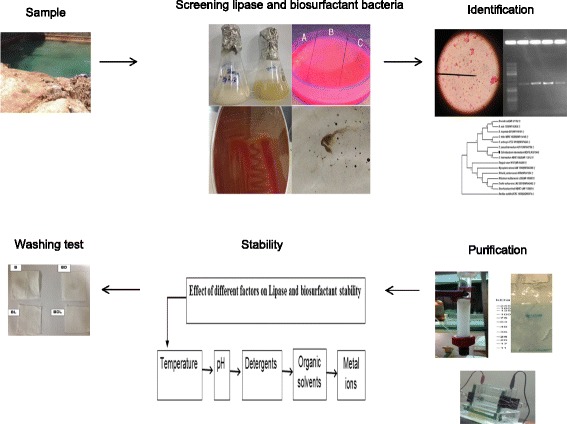

## Background

Many reports have demonstrated that scientists are interested in microorganisms which live and survive from harsh environments such as hot springs, because of their outstanding component, which is more adapted to industrial applications [1–3]. The most important metabolite produced by microorganisms is enzymes like lipase, which can serve as biocatalysts in biotransformation and other industrial processes. Additionally, surface-active agents including, biosurfactants which are produced by various types of microorganisms are widely used in detergent industry due to their eco-friendly characteristics and cost-effective production [4, 5]. Generally, the intensive applications of lipase and biosurfactant include cosmetics, pharmaceutical, agriculture and food industries [5–7]. Considering these metabolites are responsible for cleaning the oily contaminants, they have critical roles in detergent formulations. Besides, the industrial value of biological compounds strongly depends on their stability and adaptability under the production conditions [8, 9]. Therefore, lipases and biosurfactants which are able to function in the temperature range of 30–60 and pH range of 9–12 are used as the major components of enzyme-containing detergent [10, 11]. Other advantages of lipase and biosurfactant for industrial applications include biodegradability, cost-effective production and low toxicity. Since a majority of industrial production is the cost of raw materials applying bacteria which utilize substrates like agro waste and industrial byproducts counts as an economic strategy for industries [12]. For reducing the production cost of microbial metabolites, if the production condition is design based on both compounds, it would be more advantageous for manufacturers. However, since there is a constant striving for energy efficiency, especially with household appliances, the use of lower washing temperatures must be traded off against the hygiene efficacy of laundering which is considerably influenced by the temperature profile [13]. Although washing in low temperature as explained can solve energy striving problem, but laundering at low temperature can influence hygiene. Antimicrobial biosurfactant in laundry detergent can improve hygiene efficiency [14, 15].

We attempted to isolate and identify alkaline thermostable lipase and biosurfactant producing bacterium with antimicrobial activity in the same medium from Gheynarje Nir hot springs in order to use at detergent applications. In the present study, we reported *Ochrobactrum intermedium* strain MZV101 as lipase and biosurfactant producing bacteriumwhich was isolated in the presence of washing powder and considered its detergent compatibility by examining different environmental factors, metal ions, organic solvents and detergents. Finally, we were studied oil removal ability by washing test for detergent formulation.

## Methods

### Materials

Chemical compounds with their catalog number which used in this studied are DEAE- Cellulose (30477), Olive oil (75343), Sephadex G-50 column (S5897), Tween 20 (P9416), Tween-80 (P8074) and p-NPP (N2752), Sigma-Aldrich, Germany; Dialysis tubing cellulose membrane flat with 10 mm (D9277) and Rhodamine B (83689), Sigma, Germany; Millipore membrane filter 0.22 μm (GSWP02500) and 0.45 μm (HAWP04700), Merck Millipore, Germany; Acetone (100014), Ammonium sulphate (101217), Barium chloride (101716), Benzene (101783), Calcium chloride (102083), Chloroform (107024), Cobalt (II) chloride anhydrous (802540), Cyclohexane (102817), Ethanol (818760), Ethyl Acetate (100789), Hexane (104373), Isopropanol (113350), Magnesium Chloride (814733), Mercury(II) chloride (104417), Methanol (822283), Sodium deoxycholate (106504), Sodium dodecyl sulfate (817034), Toluene (108327), Triton X-100 (112298), Urea (108486) and Zinc chloride (108816), Merck, Germany. No human or animal was used in this research.

### Sampling and media preparation

Sampling was conducted from Gheynarje Nir hot spring Ardebil, Iran (latitude 38° 2′8.98″N; longitude 47°59′0.39″E). Environmental conditions like temperature and pH was recorded as 60 °C and 8.6, respectively.

The medium used for lipase and biosurfactant production was a basal salt medium as described by Schlegel 1961 with some modification [13]. This medium comprised 5 g/l yeast extract, 10 ml of olive oil, 0.2 g/l of MgSO4.7H_2_O, 0.01 g/l of FeSO_4_.7H_2_O, 0.01 g/l of CaCl_2_.7H_2_O, 1 g/l of NH4Cl, 0.5 g/l of K_2_HPO4 and 0.1 ml of trace element solution (70 mg/l of ZnCl_2_, 100 mg/l of MnCl_2_.4H_2_O, 200 mg/l of CoCl_2_.6H_2_O, 100 mg/l of NiCl_2_.6H_2_O, 20 mg/l of NaMoO_8_.2H_2_O, 26 mg/l of Na_2_SeO_3_.5H_2_O and 1 ml of 25% HCl) [13]. We used 1% olive oil as source of carbon, energy and lipase and biosurfactant stimulator. First, 10 ml samples were grown in 250 ml sterile flasks containing 80 ml of basal medium supplemented with 1% of enzyme free detergent powder (Paksan Company, Iran), each culture was adjusted to pH 8, 9 and 10 and incubated in a rotary incubator at a temperature of 60 °C at 120 rpm for 72 h. In order to make solid plate, 20 g/l agar was added to basal medium. Isolated strain was cultured on agar plate incubated at 60 °C for 48 h. Finally, a loop full of isolated strain was added to 100 ml basal medium broth and incubated at 60 °C, pH 10 and agitation speed 180 rpm for 72 h.

### Screening and isolation of lipolytic microorganism

Screening of lipase bacteria was obtained on solid plate assay for bacterial lipases according to Kouker and Jaeger’s method [16]. Based on the highest lipase specific activity, one colony was chosen for further investigations.

#### Lipase activity assay

The lipase activity was determined with p-NPP as substrate according to Winkler and Stuckmann’s [17]. Protein concentration was determined according to Lowry’s method [18].

### Screening for biosurfactant production microorganism

#### Hemolytic activity

Hemolytic assay was used as qualitative method for biosurfactant production on 5% sheep blood agar plate. The clear zone around bacteria colony was considered as biosurfactant activity [19].

#### Oil spreading assay

Oil spreading assay was measured in 25 cm plastic Petri dish as describe by Willumsen et al. Triton X-100 and water was used as the positive and the negative controls [20].

#### Drop collapsing test

Drop collapsing test was performed in to the well of a 96-well micro-plate lid according to Bodour and Miller-Maier [21]. A well without surfactant was used as negative control and Triton X-100 with 1 mg/ml concentration was used as standard surfactant for positive control [21].

#### Emulsification index (%)

Emulification index performed in a test tube as described by Bodour and Maier [21]. Triton X-100 was used as a surfactant for the positive control, and negative control was maintained without surfactant with buffer and heavy petroleum [21].

$$ EI\left(\%\right)=\frac{\mathrm{Height}\  \mathrm{of}\  \mathrm{emulsified}\  \mathrm{layer}}{\mathrm{Total}\  \mathrm{height}\  \mathrm{of}\  \mathrm{the}\  \mathrm{liquid}\  \mathrm{column}}\times 100 $$

### Antimicrobial activity

Several standard microorganisms strains like gram positive, gram negative bacteria and fungi were cultivated on nutrient agar plate at 37 °C, 24 h for bacterial and 48 h for fungi pathogens. The antimicrobial activity was investigated by disc diffusion method on MHA plate by using Bauer et al. [22] method. The diameter of inhibition zone was measured [22].

### DNA extractions, PCR amplification, sequencing and analysis

Isolated bacterial were inoculated in a medium without olive oil containing 1 g/l sodium acetate, 1 g/l sucrose and incubated in a rotary shaker at 60 °C for 48 h. Furthermore, bacteria were cultivated on a solid plate containing 1 g/l sodium acetate, 1 g/l sucrose and 15 g/l agar in order to obtain single pure colonies, and incubated at 60 °C for 24 h. DNA extraction was carried out according to the Bust n’ Grab protocol [23]. The quality of extracted DNA was evaluated using nano drop spectrophotometer readings (Thermo Scientific, Wilmington, DE). The 16S rDNA gene sequencing amplification were conduct using universal primer forward 27F (5′-AGAGTTTGATCMTGGCTCAG-3′) and primer reverse 1492R (5′-GGCTACCTTGTTACGACTT-3′). Thereafter, PCR product was purified using high pure PCR purification kit according to the manufacturer’s instructions. Nucleotide sequencing of the amplified fragments was performed with dideoxy chain termination method (SEQLAB, Germany). DNA sequences were analyzed using BLAST program (https://blast.ncbi.nlm.nih.gov/Blast.cgi) [24]. The phylogenetic tree was drawn using molecular evolutionary genetic analysis version 5.0 software (MEGA, Germany). A phylogenetic tree was constructed by using the neighbor-joining method and it was analyzed based on 16S rDNA gene sequences compared to available sequences in the GenBank [25].

### Effect of different factors on lipase and biosurfactant stability

The effect of different parameters on lipase activity was measured by incubating the extracellular enzyme with p-Nitrophenyl Palmitate as substrate for 4 h. In order to study biosurfactant emulsification index values were incubated free cellular biosurfactant in mixture of 2 ml of heavy petroleum for 24 h.

#### Effect of various pH

Lipase and biosurfactant stability was calculated in different pH range of 5–13 at 37 °C according to the same temperature of Winkler and Stuckmann’s protocol assay [17]. The buffers used in this study are (0.05 M), citrate–phosphate buffer pH 5–6, Tris-HCl buffer pH 7–9, NaHCO3-NaOH buffer pH 10–11 and KCl-NaOH buffer pH 12–13.

#### Effect of temperature

The effect of temperature was studied at a wide range from 4 to 90 °C in optimal pH 9 on Lipase and biosurfactant stability *O. intermedium* strain MZV101.

#### Effect of different metal ions

The effect of various reagents was investigated by adding reagents at a concentration of 1 mM of metal ions (BaCl_2_, CaCl_2_, CoCl_2_, HgCl_2_, MgCl_2_ and ZnCl_2_) to the enzyme and biosurfactant reaction mixture was incubated in optimal pH 10, and temperature of 60 °Ϲ [26]. We incubated the lipase enzyme for 4 h and biosurfactant for 24 h.

#### Effect of different organic solvents and detergents

The lipase enzyme and biosurfactant were incubated in the presence of organic solvent at a final concentration of 0.1% acetone, benzene, chloroform, cyclohexane, ethanol, ethyl Acetate, hexane, methanol, toluene and detergents 1% SDS, 25% Tween-20, 25% Tween- 80 and 25% Triton X-100 at pH 10 and at a temperature of 60 °C [27].

### Solvent purification lipase and biosurfactant

Lipase and biosurfactant production was carried out in same basal medium and after 72 h incubation at 60 °C. The culture broth was centrifuged at 13300×g for 10 min at room temperature. In order to achieve the best result, various precipitation methods such as ammonium sulphate with acetone [27] chilled ethanol [28] and isopropanol were studied. At the final step of each method, pellets were filtered through a sterile 0.45 μm millipore membrane. The mentioned precipitation methods for both lipase and biosurfactant were the same.

The precipitate was resuspended in 2 ml of 0.1 M Tris-HCl buffer at pH 10 and dialyzed against 0.1 M Tris buffer at pH 10 for 2 h at room temperature. The concentrated enzyme was loaded on a sephadex G-50 column (2 cm × 150 cm) according to manufacture instructions. The fraction containing protein was determined by specific activity. Purity of protein was evaluated by using SDS-PAGE gel as describe by Laemmli [29].

### Washing test

For the investigation of washing performance, white fabric cotton (5 cm × 5 cm) was degreased in boiling chloroform for 4 h and stained with 0.5 ml mixture of olive oil and benzene (100 mg/ml concentration) [11]. The white fabric was stained twice and air dried at room temperature. The stained pieces of cottons were incubated in washing solutions as mentioned in Table 1. They were incubated on 120 rpm shaker for 1 h at 37 °C. The cotton clothes were rinsed with 100 ml distilled water for 3 min at 37 °C. Olive oil was extracted from cotton fabric with petroleum ether (bp 40–60 °C) for 4 h in Soxhlet extractor [26]. The weights of olive oil were measured before and after washing for each treatment. The efficiency of oil removal was calculated by following equation:

$$ Removal\ \left(\%\right)=\frac{\mathrm{Wb}\hbox{--} \mathrm{Wa}}{\mathrm{Wb}}\times 100 $$

Wb = Weight of oil before washing.

Wa = Weight of oil after washing.

**Table 1:** Composition of washing solutions

	Volume (ml)					
Solutions	B	B + L	B + S	B + D + L	B + D + S	B + D + L + S
0.1 M Tris buffer pH = 8.5	40	40	40	40	40	40
Lipase	–	10	–	10	–	10
Biosurfactant	–	–	10	–	10	10
1% Detergent solution	–	–	–	40	40	40
Distilled water	60	50	50	10	10	–

B = 0.1 M Tris buffer pH = 8.5

L = lipase (1000 U)

S = biosurfactant (1000 U)

D = 1% Detergent solution

The stained fabrics were incubated in 250 ml flasks containing 100 ml washing solutions as mentioned below. All tests were repeated at least in triplicate independent experiments

### Statistical analysis

Experimental data were repeated at least three times. Results are reported as the mean standard deviation. The obtained data were used analyzed using repeated measure ANOVA and significant differences were determined using mauchly’s test of sphericity. The results with a *p*-value less than <0.05 were considered as significant differences. Statistically analysis was performed using IBM SPSS statistics version 22 software (IBM, USA).

## Results and discussion

### Screening and isolation of lipolytic and biosurfactant microorganism

Detergent formulations processes are performed under extremely high pH and temperature along with other addictive. The structure of thermophlic enzymes from living microorganism in hot springs are naturally stable and active at high temperatures [30]. Thermophilic enzymes from this microorganism are function at highly alkaline conditions, salinity and other harsh conditions [31]. Although all isolated bacteria are not capable to become commercially available [32]. So, screening of new lipase and biosurfactant producing bacteria are still required [33].

Our sampling site was a hot spring with 60 °C and pH 8.6. Such extreme ecosystem is one of the places in where resistant bacterial groups to harsh conditions could be found. These bacteria might produce metabolites that are suitable for industrial applications. As the favorable cleaning condition for washing powders is at high temperature and alkaline pH, therefore, having the components with high stability to temperature and pH could enhance the cleaning efficiency of detergents when applied as additive in the formulations.

Eighteen strains were isolated from culture with pH 10.In this study, we observed significant clear zone around bacteria colony. The hemolytic assay is not the specific method for screening biosurfactant producing microorganism and has limitation. Therefore, the hemolytic assay should be used as a primary method and drop collapse test, oil spreading assay and emulsification assay methods were studied to determine biosurfactant production [34].Only six strains were positive in lipase and biosurfactant. One strain was chosen for further investigations base on the fact that it had the highest emulsification index (E24 = 62.2%), and lipase specific activity of 1.8 U/mg Protein and antimicrobial activity. The isolated strains showed the positive result for drop collapse assay, oil spreading assay. Same results were reported by Nalini et al. (2013) from *Serratia rubidaea* SNAU02 with positive blood hemolysis*,* drop collapse assay and emulsification index of 52.2% [35].

### DNA extractions, PCR amplification, sequencing and analysis

**Fig. 1:**
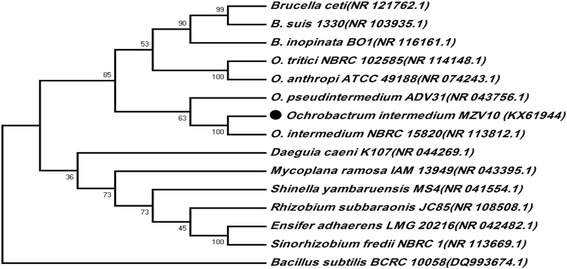
Phylogenetic tree. The Phylogenetic tree is generated on the basis of 16S rDNA gene sequence of isolated *O. intermedium* strain MZV101 using neighbor-joining method. According to 16S rDNA gene sequencing, BLAST in NCBI GenBank (access number KX619441) and strain was closely related to *Ochrobactrum intermedium*

According to 16S rDNA sequencing, biochemical and morphological tests (Table 2), the isolated strain MZV101 was identified to be closely related to *Ochrobactrum intermedium* with 99% homology via DNA BLAST in NCBI GenBank and it was recorded with the number KX619441access in NCBI*.* A phylogenetic tree of strain MZV101 based on 16S rDNA using neighbor-joining method is shown in Fig. 1 [25]. *Ochrobactrum* is a gram-negative, strictly aerobic, catalase and oxidase positive and usually single-cell [36]. *O. intermedium* 2745–2 was the first environmental strain isolated from formation water in China [33]. The optimum growth temperature for *O. intermedium* strains are 20 to 37 °C and optimum are pH 7–7.3 [37]. In this study, *O. intermedium* strain MZV101 was isolated from a hot spring at a temperature of 60 °C and pH of 8.6 for detergent application. Lipase from *Pseudomonas* sp. and *Bacillus* sp. are commonly used in detergents, also *Candida* and *Chromobacterium* [38]. They are reports regarding the production of biosurfactant from *Ochrobactrum anthropi* strain, *Ochrobactrum* sp. 1C, *Ochrobactrum anthropi* strain 2/3, *Ochrobactrum sp*. strain BS-206 (MTCC 5720) [2, 39–41]. However, Mishra et al. reported biosurfactant and lipase production from *O. intermedium* strain P2 [42].

**Table 2:** Phenotypic characteristics of isolated bacteria *O. intermedium* strain MZV101

Test	*O.intermedium* Strain MZV101	*O.intermedium* *strain LMG3301*
Growth at 37 °C	+	+
H_2_S production/Utilization of:		
Glycine	+	+
D-Alanine	+	+
L-Aspartate	+	+
D-Arabinose	+	+
L- Arabinose	+	+
Mannitol	+	+
Sorbitol	+	+
D-Lyxose	+	+
D-Fructose	+	+
Mannitol	–	–
D- Sorbbitol	+	+
Glycerol	–	–
Sucrose	+	+
Hydrolysis of:		
Gelatin	–	–
Urease test after 24 h	–	–
Urease test after 48 h	–	–
Utilization of:		
Citrate	+	+
Sensitivity to antibiotics		
Amoxicillin (25 mg)	R	R
Colistin (50 mg)	R	R
Chloramphenico (30 mg)	R	R
Tetracycline (30 mg)	R	R

Morphological, physiological and biochemical characteristics of *O. intermedium* strain MZV101 had comparison with *O. intermedium* strain LMG3301. Results are scored as (+) positive, (−) negative and (R) resistance. Data were obtained from velasco et al. study [36]

### Effect of different factors on lipase and biosurfactant stability

#### Effect of pH

**Fig. 2:**
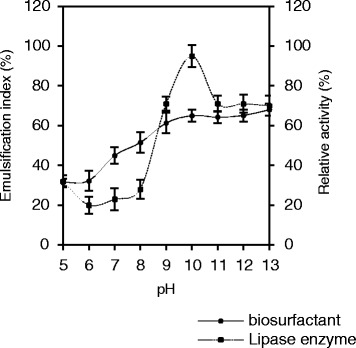
Effect of pH on lipase and biosurfactant stability of *O. intermedium* strain MZV101.The test were studded in pH 5–13 in temperature 37 °C which incubated 4 h for lipase and 24 h for biosurfactant. Results were represented as mean ± S.D. and all tests are examined at least in triplicate independent experiments. Error bar has indicated standard deviations shown in all figures

High pH could modify protein structure and consequently changes the enzyme activity. The optimum lipase bacteria activity reported to be over ranges of pH 5–9 [43]. Generally, laundering performed at alkaline conditions, thus alkaline lipase were prefer for laundry detergent [33]. So, enzyme function at high pH is essential in detergent formulation [44]. *O. intermedium* strain MZV101 lipase maintains its activity and stability at pH 5–13 with maximum activity at pH 9 at 37 °C (Fig. 2). Lipase enzyme *O. intermedium* strain MZV101 has 80% stability at pH 10–13 and minimum stability between pH 6–8. The enzyme also remains stable after 24 h in all indicated pH range at room temperature. Chen et al. have declare that alkaline lipase from *Achetobacter radioresistens* was stable in optimum pH 10.0 at 30 °C and as a good potential for detergent application [45]. This report is in agreement with our results. Accordingly, other studies revealed high relative activity at lower pH from our obtained data in this study. For instance, Golaki et al. (2015) have been reported 70% relative activity of lipase 3646 from thermophilic indigenous *Cohnella* sp. A01 was stable in the pH range of 7 to 9 and maximal activity was obtained at pH 8.5 [46].

Biosurfactant with high stable ability in the presence of alkaline condition is required for the detergent additive because pH of laundry is generally between ranges of 9.0–12.0 [47, 48]. Many reports have been revealed the important role of bacteria biosurfactant as laundry detergents additives [12, 47, 49]. High stability was observed from pH 10–13 (E_24_ average = 65.58%), on the other hand, emulsification index values decreased from pH 5–6 (E_24_ average = 31.84%) (Fig. 2).These results showed that emulsification index values are accompanied with pH. These results are in agreement with those by Shavandi et al. who reported that emulsification activity was sensitive to pH variations [50]. From our same species, Ferhat et al. reported that biosurfactant from *Ochrobactrum* sp. 1C the highest (E24 average = 95%) at pH 7 to 11 was observed, while emulsification values decrease to (E24 = 70.58%) at pH 4 and temperature 37 °C [39]. Bhattacharya et al. have declaim that biosurfactant from *Ochrobactrum* sp. C1 the highest emulsification index values (E24 = 69.42%) were observed at low pH 7.3 and temperature 36.4 °C [51].

#### Effect of temperature

**Fig. 3:**
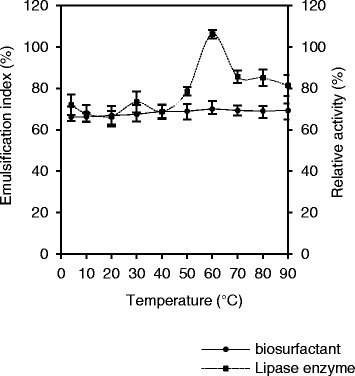
Effect of temperature on lipase and biosurfactant stability of *O. intermedium* strain MZV101. Experiments were measured in temperature 10–90 °C in optimal pH 10 which incubated 4 h for lipase and 24 h for biosurfactant

Lipase enzyme with activity and stability at the wide range of temperature is favoring characteristic for detergent applications, at high temperatures due to their responses provides fewer microbial contamination threats, high solubility of substrates and low viscosity of the reaction elements [52]. At low temperature can accomplish with synthetic unstable compounds and improves oil elimination from fabric; consequently, reduces energy consumption [52–54]. According to test results, lipase of *O. intermedium* strain MZV101 had remained active and stable at temperature range of 4–90 °C (Fig. 3). The lipase of *O. intermedium* strain MZV101 remains 80% active at 70–90 °C and with an optimal activity at 60 °C for 1 h at pH 10. In contrast to other lipases, lipase enzyme of *O. intermedium* strain MZV101 remained active even after 4 h at 60 °C [5]. Similarly, 60% relative activity for lipase 36,464 from thermophilic indigenous *Cohnella* sp. A01 at 60 °C and pH 10 for 180 min has been reported [46]. Lipases from *Bacillus megaterium* AKG-1 and *Acinetobacter* sp. have reported to be in the optimum temperatures range of 50–60 °C [55, 56]. Recently, García-Silvera et al. have described that lipase stability from *Serratia marcescens* wild type and three mutant strains in temperature over range of 5 to 55 °C with an optimum in temperature 50 °C and stable at pH 6 to 10 with optimum pH 8 for 1 h were obtained for detergent formulation and biodiesel [52]. Comparably with our results, Bora et al. have mentioned that the lipase from *Bacillus* sp. DH4 with optimum activity at temperature 60 °C and pH 9 as an additive for detergent formulation [57]. Cherif et al. have explained a high alkaline lipase produced by *Staphylococcus* sp. strain ESW the optimum activity was at temperature 60 °C and pH 12 and it was an ideal choice in detergent formulations [24].

Nevertheless, its emulsification index values increased with temperatures between 4 °C (E24 = 66.25%) to 60 °C (E24 = 70.41%), and this value remain stable between temperatures of 70–90 °C (E24 average = 69.34%) (Fig. 3). According to results, biosurfactant of *O. intermedium* strain MZV101 at wide range of temperature (4–90 °C) had remained stable and had no significant effect on emulsification index. Ferhat et al. reported that biosurfactant produced by *Brevibacterium sp.* 7G, it emulsification index values remain stable between 20 and 100 °C [39]. Mukherjee reported that biosurfactant produced by *Bacillus subtilis* DM-03 and DM-04 have thermal stability at pH 7–12 and 80 °C for 60 min, also compatibility and stability with commercial laundry detergents [48]. Comparable with our data, Sajna et al. mentions that biosurfactant from *Pseudozyma sp.* NII 08165 as an additive for laundry detergent at pH 8–12 and temperature 80 °C was stable for 2 h [47]. It has been described that biosurfactant from *Bacillus subtilis* PF1with good stability at pH 6–11 and temperature 30–60 °C and emulsification index (E24 = 100%) at pH 10–11 and 30 °C as laundry detergent additive [12]. In contrast to our data, other studies found stable biosurfactant as detergent additive in low pH and temperature. For instance, Hirata et al. [59] have reported that glycolipid biosurfactant from *Candida bombicola* ATCC22214 was active at temperature 20 °C and pH 8.94 as biodegradable low-foaming surfactants with high detergency ability [58].

#### Effect of different metal ions

Lipase enzyme with detergent compatibility acts differently in presence of metal ions [54]. Various studies have demonstrated that the activity of lipase enzyme depends on the presence of Ca^2^+, our study shows that lipase *O. intermedium* strain MZV101 can be activated in the absence of Ca^2^+ [49, 59]. Lipase of *O. intermedium* strain MZV101 was 55% inhibited by Mg^2^+ and 33% by Hg^2^+ and Ba^2^+, while it was activated toward Co^2^+ (Table 3). Kanwar et al. (2006) have reported lipase from *Bacillus coagulans* MTCC-6375 an average relative activity 112.93% on the presence of 1 mM of Mg^2^+, Ca^2^+ and Hg^2^ +, while activity decreases to 39.7% toward 1 mM of Co^2^+ [60]. Sekhon et al. have explained that an extracellular lipase isolated from *Bacillus megaterium* AKG-1 in existence of metal ions Co^2^+, Ca^2^+ and Mg^2^+ stimulated the enzyme activity to 276, 325, 250% respectively, whereas Hg^2^+ and Zn^2^+ inhibits lipase enzyme activity to 58% [56].

**Table 3:** Effect of metal ions on biosurfactant and lipase stability of *O. intermedium* strain MZV101

Metal ions	Emulsification index (%)	Relative activity (%)
Control	100	100
BaCl_2_	95.99	78.8
CaCl_2_	96.27	97.41
CoCl_2_	95.79	114.58
HgCl_2_	95.49	81.52
MgCl_2_	94.01	48.68
NaCl	94.38	87.70
ZnCl_2_	95.67	80.70

The experimental were tested with final concentration of 1 mM on lipase and biosurfactant stability of *O. intermedium* strain MZV101 in optimal pH 10, and temperature of 60 °C incubated respectively for 4 h and 24 h. In this study, results are expressed as mean ± S.D. and all tests were examined at least in triplicate independent experiments

High stability of biosurfactant *O. intermedium* strain MZV101 was observed in the presence of Ca^2^+, Mg^2^+, Zn^2^+, Hg^2^+ and Co^2^+ (E_24_ average = 95.53%) (Table 3). In this study, results revealed that other metal ions had no significant effect on enzyme and biosurfactant stability (Table 3). It is a very important factor for detergent formulate that enzyme and biosurfactant stay stable at presence of metal ions [61]. As pointed by Kanna et al. that biosurfactant from *Pseudomonas putida* MTCC 2467 after 48 h incubation in presence of metal ions Ca^2^+, Mg^2^+ and Hg^2^+ with 20 (g/l) concentration, it activity was less than control and no significant difference in effect between metal ions [62]. Maneerat et al. reported that crude biosurfactant from *Acinetobacter calcoaceticus* subsp. *anitratus* SM7 also remained stable in the presence of low concentration of Ca^2^+ and Mg^2^+ metal ions [61]. The findings of the present study are in line with the results in the preceding paragraph. Ochoa-Loza et al. have observed lower activity by biosurfactant from *Pseudomonas aeruginosa* ATCC 9027 in the presence of Zn^2^+, Ca^2^+, Hg^2^+ Mg^2^+, and Co^2^+ with 0. 5 mM concentration at pH 6.9 and room temperature incubation for 2 h [63]. This is in inconsistent with our obtained data in this research.

#### Effect of different organic solvents and detergents

Lipase enzyme with removal ability could not be effective as detergent addictive component [11]. Functional alkaline lipase in the presence of other detergent and ingredients are necessary specifications for detergent application [32]. Toida et al. have reported that the activity of lipase dramatically reduced in the presence of 1% SDS [64]. In contrast to other reports, lipase produced by *O. intermedium* strain MZV101 not only was stable in the presence of 1% SDS, but even showed 30% increase of relative activity. Recently, the reports have shown that addition of Tween-20 increase lipolytic activity of some lipase enzymes. In this study, the lipase enzyme has about 70–80% in the presence of Tween- 80, benzene and Chloroform as seen in Table 1 [64]. Lipase enzyme activity has exhibited an average activity of 117.03% in the presence of ethyl acetate, toluene and cyclohexane (Table 4). Lipase from *Pseudomonas fluorescens* P21 has been found to be stable toward toluene, benzene, cyclohexane and hexane with residual activity 66.7, 68, 80 and 94.1% respectively after incubation for 2 h [65]. These results are in line with our obtained data in this research. Lipase enzyme strain MZV101 was shown 35.11% relative activity in existence of Triton X-100. Several authors have been reported solvent tolerant lipases produced by *Pseudomonas* sp. and *Burkholderia* sp. [37, 65]. In contrast to our results, Rathi et al. have described that alkaline lipase from *Burkholderia cepacia* RGP-10 for detergent formulation remains 80% active in presence Triton X-100, while 33, 57 and 40% relative activity toward SDS, Tween-20 and Tween-80 respectively at 37 °C and pH 11 for 1 h [37]. 80–75% inhibition was shown toward acetone, ethanol and methanol after incubation for 4 h. Relatively close to our observations, Bose et al. have explained that 25, 50 and 75 (*v*/v%) methanol caused approximately 90–97% inhibited lipase activity from *Pseudomonas aeruginosa* AAU2 activity after 24 h and 48 h of incubation [66]. Equally, Lipase from *Pseudomonas fluorescens* P21 has been reported to be unstable toward acetone and methanol [65].

**Table 4:** Effect of 0.1% organic solvents and 25% detergents on biosurfactant and lipase of *O. intermedium* strain MZV101 stabilities

Organic solvents/detergents	Emulsification index (%)	Relative activity (%)
Control	100	100
Acetone	94.59	20.00
Benzene	88.23	77.46
Chloroform	88.98	44.01
Cyclohexane	87.83	112.67
Dimethyl	82.45	88.02
Ethanol	92.99	23.05
Ethyl Acetate	91.21	107.18
Hexane	86.75	101.01
Methanol	93.23	25.07
Toluene	87.56	131.26
1% SDS	98.65	122.53
Tween-20	92.34	117.74
Tween- 80	91.23	80.98
Triton X-100	85.30	35.11

The tested were studied on lipase stability of *O. intermedium* strain MZV101 in optimal pH 10 and in optimal temperature 60 °C incubated for 4 h and same mentioned condition was used for biosurfactant *O. intermedium* strain MZV101 incubated for 24 h. Results were represented as mean ± S.D. and all tests are examined at least in triplicate independent experiments

The maximum emulsifying activity values were obtained by Acetone, Methanol and Ethanol (E_24_ average = 93.60%). A slight decrease in emulsification activity of biosurfactant *O. intermedium* strain MZV101 was observed at 1% SDS (E_24_ = 97.89%), 25% Tween-20 (E_24_ = 94.34%) and 25% Tween-80 (E_24_ = 91.23%). 25% Triton X-100 shows the most inhibition of biosurfactant activity (E_24_ = 85.31%) (Table 1). The result is in comparable with that of Ben Ayed et al. (2013) who have declare that biosurfactants from *Bacillus mojavensis* A21 in presence of SDS and Tween-80 with concentration 5 mg/ml at 20 °C incubated for 24 h was observed to be (E24 average = 71%) [67]. In another study, Kim et al. have declare that biosurfactants from *Nocardia* sp. L-417 was stable with maximum emulsification activity in presence of Triton X-100 and less activity by Tween-80 and SDS at 30 °C and pH 6 for 50 min [68]. No significant differences were observed between emulsifying activity values (E_24_ average = 87.43%), inhibition by the addition of Benzene, Chloroform, Cyclohexane and Hexane. Darvishi et al. have explain that a new microbial consortium of *Enterobacter cloacae* and *Pseudomonas* sp. ERCPPI-2 with emulsifying activity values at the presence of hexane (E_24_ = 43.4%) and cyclohexane (E_24_ = 57.5%) was observed [69]. Our study shows an inferior value with hexane and cyclohexane by *O. intermedium* strain MZV101.

### Solvent purification lipase and biosurfactant

Lipase enzyme in detergent application dose not required high level of purity [70]. Abdel-Mawgoud et al. (2010) reported the purity level of biosurfactant related to final product application [71]. The biosurfactant and lipase of *O. intermedium* strain MZV101 were precipitated with three methods; 30–80% ammonium sulphate with acetone, ethanol and isopropanol precipitation. In order to precipitate the lipase enzyme, we attempted all mentioned methods. Enzyme precipitation failed except isopropanol which is even less common method for nucleic acid precipitation [72]. Various concentration of isopropanol from 50 to 90% was investigated. Although precipitation by organic solvents required 80–90% alcohol concentration to precipitate lipase enzyme [73], we obtain best result by 60% chilled isopropanol with (56.30 U/mg and yield 51.71%) (Table 5).

**Table 5:** Extraction of biosurfactant and lipase from *O. intermedium* strain MZV101

Precipitation methods	Emulsification index (%)	Biosurfactant yield (%)	Specific activity (U/mg)	Lipase enzyme yield (%)
Ammonium sulphate	60.11	48.04	0.00	0.00
Acetone	62.87	38.21	0.00	0.00
Ethanol	70.99	50.32	0.00	0.00
Isopropanol	0.00	0.00	56.30	51.71
dialysis	69.45	48.63	55.00	49.62

Results of solvent extraction and dialysis biosurfactant and lipase of *O. intermedium* strain MZV101 were represented as mean ± S.D. All tests were examined at least in triplicate independent experiments

Even though these results differ from some earlier studies, Ameri et al. found that 80% pre-chilled ethanol with 39.8% yield was the best method to precipitate thermoalkalophilic lipase from bacterial strain *Bacillus atrophaeus* FSHM2 [74], and also Raza et al. (2017) found that 80% ammonium sulfate with 5.3 fold was the most suitable method to precipitate the lipase from *Staphylococcus aureus* for detergent industry [75], we found that this method can completely inactive the lipase from *O. intermedium* strain MZV101.

Isopropanol was unable to precipitate biosurfactant *O. intermedium* strain MZV101.The best result (E_24_ = 70.99% and yield = 49.32%) was obtained from ethanol and also good results were obtained with ammonium sulphate (E_24_ = 60.11% and yield = 48.04%) and acetone (E_24_ = 62.87% and yield = 38.21%) (Table 5). It has been reported that biosurfactant from *Nocardia* sp. L-417 was stable by extraction of ammonium sulphate fractionation and chilled acetone in two primary purification steps [68]. The best results obtained by cold ethanol precipitation (E_24_ = 70.99% and yield = 50.32%). Same result was reported by Ramasamy et al. for cold ethanol precipitation of the biosurfactant produced by biosurfactant *Ochrobactrum anthropi* MP3 [76]. Our experiments corroborate with previous results Salleh et al. who explain that organic solvent extraction was found as the best giving and the highest recovery at 89.70% (*w*/*v*) purification method for biosurfactant produced by *Pseudomonas aeruginosa* USM AR-2. Additionally, they were obtained good results with ammonium sulphate/acetone method precipitation [77].

**Fig. 4:**
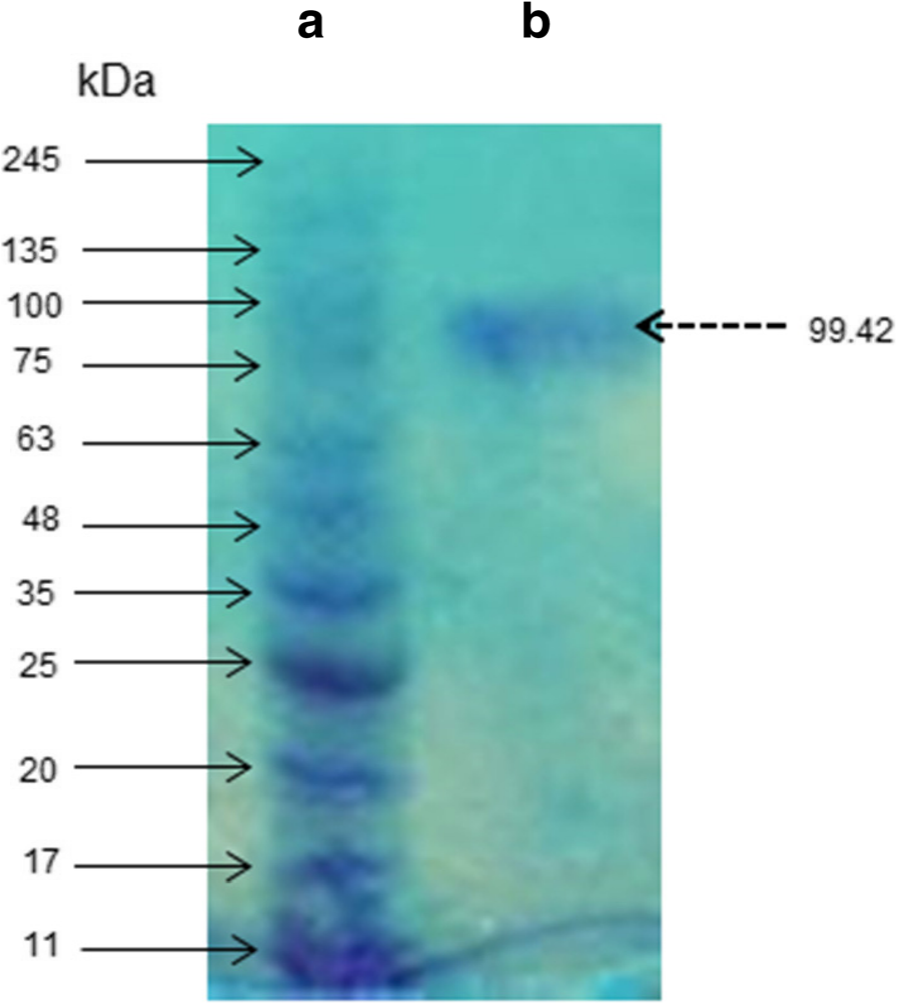
SDS-PAGE gel of Lipase enzyme *O. intermedium* strain MZV101. (A) standard marker proteins, (B) Single protein band become visible at 99.42 kDa on 12% sodium dodecyl sulfate-polyacrylamide gel after stained with coomassie blue G 250

Both enzyme and biosurfactant purification were continued by short dialysis against 0.1 M Tris buffer pH 9 and insignificant purity of biosurfactant and lipase was observed (Table 5). At the final step of lipase purification, the following results were obtained after the investigation of suitable column: DEAE- Cellulose column with a recovery of 5.241%, 2.649 fold, and G-10 Sephadex column with a recovery of 7.082%, 3.038 fold. Finally, the best result with a recovery of 48.452% and 19.095 fold was obtained from G-50 Sephadex column. The single protein band molecular mass was estimated on SDS-PAGE to be 99.42 kDa (Fig. 4). Comparable with our results, Kanwar et al. (2006) have mentioned high molecular mass of approximately 103 kDa on SDS-PAGE for lipase from *Bacillus coagulans* MTCC-6375 [60]. Furthermore, Dosanjh et al. have reported higher molecular mass 112 kDa from *Bacillus* GK 8 [78]. In contrast, Kumar et al. have describe lower molecular mass 31 kDa from *B. coagulans* BTS-3 [79] and Huang et al. have published protein mass 62-kDa lipase from *Geotrichum marinum* [80].

### Antimicrobial activity

Antibacterial detergents experimentally were proved to have bacteristatic activity, inhibit their growth and kill bacteria at a specific concentration [81]. Antimicrobial biosurfactant in detergents are very effective against some Gram-positive and Gram-negative bacteria specially, it could be very helpful against multi-drug-resistant pathogens [37, 82], and on the other hand, it can cause antimicrobial resistances in bacteria. Gharaei-Fathabad et al. reported that biosurfactant from *B. circulans* has antimicrobial activity against Gram negative and Gram positive pathogens and semi pathogenic microbial strains including MDR strain [83]. In this study, purified biosurfactant of *Ochrobactrum intermedium* strain MZV101 were used. The result of antimicrobial activity was investigated against Gram-positive and Gram-negative bacteria by measuring the zone of inhibition (Table 6).

**Table 6:** Biosurfactant as an antimicrobial agent produced by *O. intermedium* strain MZV101

Microorganism	Zone of inhibition diameter (mm)
*Gram positive*	
*Bacillus subtilis* (ATCC 465)	3
*Bacillus cereus* (ATCC11778)	4
*Enterococcus faecalis* (ATCC11778)	3
*Staphylococcus aureus* (ATCC 25923 s)	2
*Streptococcus pyogenes* (ATCC 8668)	4
*Gram negative*	
*Escherichia coli* (ATCC 25922)	14
*Pseudomonas aeruginosa* (ATCC 85327)	10
*Proteus vulgaris (*ATCC 13315)	12
*Salmonella typhi* (PTCC 1609)	11
*Fungi*	
*Aspergillus niger* (N 402)	1
*Candida albicans* (ATCC 10231),	0.5
*Klebsiella pneumoniae* (ATCC 10031)	1.5
*Saccharomyces cerevisiae* (BY 4743)	1

MHA plate was incubated at 37 °C, 24 h for bacterial and 48 h for fungi pathogens and the diameter of inhibition zone was measured [22]

Higher antimicrobial activity with diameter of 10–14 mm clear zone was observed against the Gram-negative bacteria. The highest inhibition of zone were obtained with *Escherichia coli* (ATCC 25922) (14 mm), *Proteus vulgaris (*ATCC 13315) (12 mm) and *Salmonella typhi* (PTCC 1609) (11 mm). Biosurfactant of *O. intermedium* strain MZV101 demonstrated a weak antimicrobial activity with 2–4 mm diameter of clear zone against Gram-positive bacteria, and also the same result was observed against fungi. Noparat et al. have reported high antimicrobial activity against Gram-positive and lower activity against Gram-negative of biosurfactant produced by *Ochrobactrum anthropi* strain 2/3 against several pathogens and this is in contrast to the results achieved in our study from biosurfactant of *O. intermedium* strain MZV101 [41]. The inhibition of zone biosurfactant by isolated bacteria *O. intermedium* strain MZV101 against *S. aureus* (ATCC 25923 s) and *Pseudomonas aeruginosa* (ATCC 85327) were recorded to be in respect 2 mm and 15 mm. Biosurfactant from *Ochrobactrum* sp. 1C has reported to obtained (11.1937 mm) inhibition zone with *S. aureus* (ATCC 9144) and (15.9468 mm) with *P. aeruginosa* (ATCC) [39].

### Washing test

The washing test of the lipase and biosurfactant *O. intermedium* strain MZV101 isolated in the present study was evaluated by their ability to remove olive oil stain from cloth. The solution of 1% detergent and Tris buffer could only remove 16.11% and buffer solution alone had insignificant removal ability. At alkaline condition, fatty acids were more easily eliminate from fabric but they stay on fabric because they are unable to saponify by the alkaline solution. Thus, by addition of lipase enzyme they could remove easily from fabric [15]. With the combination of lipase, detergent and buffer, high oil removal of 76.34% was observed and also the presence of lipase *O. intermedium* strain MZV101 without detergent shows 67.63% olive oil removal. Thirunavukarasu et al. (2008) had reported that the percentage of olive oil removal lipase produced by *Cryptococcus* sp. S-2 with other detergents from fabric was higher than 21.1% [65]. Grbavčić et al. reported good oil stain removal with the addition of lipase from *P. aeruginosa* in washing formulation [11]. It has been demonstrated that combination of extracelluar lipase from *Micrococcus* sp. ML-1 with different commonly used detergents could enhance the removal of greasy stains from textile [84]. Also, lipase from *Ralstonia pickettii* has been reported in the presence of detergent improves the removal of oil by 24–27% over treatment with detergent alone [15].

An oil removal about 60.11% was obtained in a solution containing buffer solution and isolated biosurfactant and also less olive oil removal of 23% was observed in solution without biosurfactant *O. intermedium* strain MZV101. Recently, it has been reported that by additional of CLP biosurfactants from *B. subtilis* strain DM-04 could enhance 9–12% elimination of oily stain from fabric [48]. Additionally, Jain et al. have mentioned that biosurfactant from an alkaliphilic bacterium *Klebsiella* sp. strain RJ-03, it alone removed 80% oil and with various laundry detergents have enhanced oil removal to (17–20%) which improved the washing performance [85].

Biosurfactant by reducing surface tension along with other enzymes is able to remove better oily stains [7]. The best result of 82.33% oil removal was obtained in combination of Lipase, biosurfactant, detergent and buffer. Hence, from the results of washing test, it can be concluded that lipase and biosurfactant *O. intermedium* strain MZV101 with good oil removal from white cotton can use as addictive washing component in laundry detergents. Same results have reported by Bhange et al. which biosurfactant and enzyme along with other detergent component could improve the stain removal efficiency [12].

## Conclusion

In this study, as main goal we demonstrated that enzyme and biosurfactant produced by isolated bacteria *Ochrobactrum intermedium* strain MZV101 had high pH and temperature stability and they remain stable at presence of other metal ions and organic solvent.

The enzyme can be activated in the absence of Ca^2^ + .They show stability in the presence of detergents and lipase enzyme activity had increased towards of 1% SDS.

Hence, *Ochrobactrum intermedium* strain MZV101 with strong oil removal capability and also antimicrobial characterization is promising candidate for laundry detergent. Lipase and biosurfactant production by bacteria in a single low cost medium, fast production with good yield not only could solve economic problems but also eco-friendly detergent additives can protect our environmental.

During our study, we found isolation of microorganism in washing powder could be an efficient method for isolating compatible strain for detergent application, and also isopropanol as a new alternative organic solvent for lipase enzyme precipitation instead of usual agents used before. Our aim was to precipitate lipase and biosurfactant with same suitable method to save money and time in detergent application; therefore, this step required further investigation.

## Abbreviations

BLAST: Basic Local Alignment Search Tool
DEAE- Cellulose: Diethyl Aminoethyl –Cellulose
kDa: Kilodalton
MHA: Muller-Hinton Agar
NCBI: National Center for Biotechnology Information
p-NPP: para-Nitrophenyl Palmitate
SDS-PAGE: Sodium Dodecyl Sulfate – Polyaryl Amidee Gel Electrophoresis
